# Preparation and Properties Study of CsPbX_3_@PMMA Luminescent Resin

**DOI:** 10.3390/mi15091150

**Published:** 2024-09-13

**Authors:** Xinqiang Ma, Shengying Fan, Wenwen Yang, Jiajie Wei, Xiaolei Wang, Jincheng Ni, Wei Cheng, Qinhe Zhang

**Affiliations:** 1 Key Laboratory of High Efficiency and Clean Mechanical Manufacture of Ministry of Education, School of Mechanical Engineering, Shandong University, 17923 Jingshi Rd., Jinan 250061, China; 2 Laser Institute, Qilu University of Technology (Shandong Academy of Sciences), Jinan 250104, China; 3 Shandong Qiangyuan Laser of SDIIT Ltd., Liaocheng 252000, China; 4 CAS Key Laboratory of Mechanical Behavior and Design of Materials, Department of Precision Machinery and Precision Instrumentation, University of Science and Technology of China, Hefei 230027, China

**Keywords:** CsPbX_3_ perovskite, luminescent resin, light curing, methyl methacrylate

## Abstract

Perovskite as an emerging semiconductor luminescent material has attracted widespread attention due to its simple preparation, high luminescence quantum yield, high color purity, tunable spectrum, and ability to cover the entire visible light band. However, due to the influence of water or other highly polar solvents, oxygen, temperature, and radiation, perovskite nanocrystals will aggregate or collapse in the lattice, eventually leading to luminescence quenching. This study starts from the postprocessing of perovskite, uses methyl methacrylate as the monomer and TPO as the photoinitiator, and encapsulates the perovskite powder prepared by the hot injection method through ultraviolet light initiation. A method is proposed to improve the luminescence and crystal structure stability of perovskite. By eliminating the influence of environmental factors on perovskite nanocrystals through the dense structure formed by organic polymers, the resistance of perovskite to strong polar solvents such as water will be greatly improved, and it has great potential in the protection of perovskite. Finally, by changing the proportion of halogen elements in the perovskite resin to change the color of the luminescent resin, a fluorescent coating emitting light in all visible light bands is prepared. Fluorescent coatings are widely used in life and industry fields such as plastics, sol, and paper.

## 1. Introduction

Perovskite was first discovered by the German scientist Gustav Rose during a geological survey. To commemorate the Russian geoscientist of the same name, it is named perovskite [1]. Early perovskite materials were purely inorganic materials. Later, researchers used methylamine molecules to produce organic–inorganic hybrid perovskite materials. As an emerging semiconductor luminescent material, perovskite nanocrystals have attracted widespread attention due to their simple preparation, high luminescence quantum yield, high color purity, tunable spectrum, and ability to cover the entire visible light band [2,3,4]. Among them, the two most widely studied metal halogen perovskite systems are methylamine perovskite and cesium perovskite nanocrystals, which have the best optical properties in the green band and have higher fluorescence efficiency and better stability than other bands under the same preparation conditions.

With the improvement and optimization of perovskite synthesis methods, the optical quality and stability of the above two types of green light perovskite nanocrystals have been increasingly improved. In previous studies, perovskite nanocrystals with controllable shapes and compositions were prepared by thermal injection, solvothermal, ultrasonic, room temperature precipitation, and chemical vapor deposition [5,6,7,8]. Due to different synthesis conditions or functional ligands, the luminescence light quantum yield is different. The luminescence light quantum yield of perovskite nanocrystals prepared by the high-temperature injection method reaches more than 90% [9]. The high-temperature injection method involves injecting one precursor solution into another preprepared precursor solution, reacting them in a high-temperature inert gas environment, and forming crystals through rapid cooling. However, this method has a high cost and a low preparation efficiency [10,11]. Precipitation synthesis at room temperature mainly utilizes the precursor, which has good solubility in polar solutions (dimethyl sulfoxide, DMSO, or N, N-dimethylformamide, DMF), and poor solubility in a weakly polar solvent (toluene). Thus, perovskite nanocrystal particles are obtained by rapid recrystallization [12]. However, due to the small ionic radius of chloride ions, they are not enough to support the stable octahedral structure. In addition, in the synthesis of iodide at room temperature, the crystal phase easily undergoes a phase transition, and the perovskite nanocrystals are also unstable. Therefore, it is difficult to synthesize cesium lead chloride perovskite and cesium lead iodide perovskite at room temperature [13].

At present, the synthesized perovskites are injected by strong electricity injection and high energy radiation, and most of them are stored in organic solutions (toluene, n-hexane, etc.), while most applications of perovskites use perovskite nanocrystals after centrifugation, which makes CsPbX_3_ susceptible to interference from external environmental factors (e.g., oxygen, water, strong polar solvents, and strong ultraviolet rays). The synthesized perovskites are widely used in LEDs, displays, and scintillators, among others [14,15,16]. To change the dispersion medium of the perovskite, the perovskite is dispersed in organic polymers or supramolecular gels to isolate water or oxygen in the air to preserve and transport the perovskite, which will also enhance the application potential of the perovskite [17]. Organic polymer materials are formed by one or several molecules or molecular groups bonded by covalent bonds. In nature, organic polymer materials widely exist in the form of fiber, protein, and natural rubber, which have the advantages of light texture, abundant raw materials, easy processing, excellent performance, and wide application [18,19]. The combination of the perovskite and the polymer has broad application prospects. Li Yingchun et al. used the organic–inorganic hybrid perovskite CH_3_NH_3_PbBr_3_ as the luminescent source and prepared the perovskite in a polymethylmethacrylate (PMMA) polymer [20]. The method greatly prolongs the service life of the perovskite and effectively improves the resistance of the perovskite to water. However, the preparation method uses a relatively complex reversible addition–fragmentation chain transfer technology, which significantly reduces the fluorescence quantum yield of the perovskite.

In summary, all inorganic perovskite nanocrystals are synthesized by a high-temperature injection method, and the synthesized perovskite nanocrystals are encapsulated in organic polymer materials (e.g., PMMA). The encapsulation of the perovskite into PMMA has the following advantages: (1) Methyl methacrylate (MMA) is a polymer monomer and will not have an obvious quenching effect on the perovskite, and the perovskite nanocrystals dispersed in MMA still have strong luminescence after being stored for 12 h; (2) The photoinitiator (2, 4, 6-trimethylbenzoyl phosphine oxide, TPO) forms free radicals under ultraviolet irradiation, and the photoinitiation rate could be adjusted by changing the mass fraction of the initiator in MMA. In the polymerization process, the initiator system with a smaller mass fraction is selected to avoid an intense heat release. (3) PMMA formed by the polymerization of MMA has good light transmittance and a compact structure, which can protect the perovskite and improve the resistance of the perovskite to water and oxygen.

## 2. Materials and Methods

### 2.1. Synthesis of CsPbX_3_ Nanocrystals

All inorganic perovskite nanocrystalline CsPbBr_3_ was synthesized by the traditional high-temperature injection method. First, 0.25 mmol cesium carbonate (Meryer, Shanghai, China) (814 mg) was weighed and added to 40 mL octadecene (Sinopharm, Shanghai, China), vacuumized and heated to 120 °C to fully eliminate oxygen in the medicine, and then heated to 150 °C in a nitrogen environment to fully dissolve it, thus obtaining pure cesium oleate solution (the prepared cesium oleate was sealed and preserved after each use and heated to the desired temperature).

A total of 0.188 mmol (69 mg) of lead bromide (Meryer, Shanghai, China) was placed in a 50 mL three-mouth flask, 5 mL of octadecene (Sinopharm, Shanghai, China) was added, 0.5 mL oleamine (Energy Chemical, Shanghai, China) and 0.5 mL oleic acid (Energy Chemical, Shanghai, China) were added, and the mixture was stirred and heated in a vacuum environment to 120 °C until the lead bromide was completely dissolved. Then, the oxygen in the container was drained by circulating nitrogen filling and vacuuming, and then it was heated to 180 °C in nitrogen. Then, 0.4 mL of cesium oleate solution heated to 150 °C was quickly injected into the lead bromide octadecene solution at 180 °C. After adding the cesium oleate solution for 5 s, the solution had a weak green luminescence. The lead bromide solution with cesium oleate added was quickly put into the ice–water mixture at 0 °C. When the system was completely cooled, it was observed that the solution had a strong green luminescence under ultraviolet light irradiation. The solution was poured into a 50 mL centrifuge tube and centrifuged at 10,000 rpm for 15 min. The supernatant was discarded, and the precipitate was dispersed into toluene, which was fully crushed by an ultrasonic machine (KQ-250DE, Kunshan, China) and centrifuged at 10,000 rpm for 15 min (Cence H1850, Changsha, China). After centrifugation, the CsPbBr_3_ perovskite precipitate and supernatant were obtained. Under ultraviolet irradiation (Shimadzu, Kyoto, Japan), the precipitate and supernatant had strong green luminescence. For the preparation of CsPbCl_3_ and CsPbI_3_ perovskite nanocrystals, one only needs to change the halogen type of lead halide, and the other conditions are the same as for CsPbBr_3_.

### 2.2. Synthesis of CsPbX_3_@PMMA Luminescent Resin

Methyl methacrylate (MMA) (Energy Chemical, Shanghai, China) was heated and reevaporated at 120 °C, and the inhibitor was removed. We added 2.5 mL of resteamed MMA into five test tubes, and then we added 7.3 mg, 21.1 mg, 36.5 mg, 51.1 mg, and 73.2 mg TPO (mass fraction was 0.1%, 0.3%, 0.5%, 0.7%, and 1%, respectively) (Aladdin, Shanghai, China) into each test tube. The polymerization was initiated with a 450 nm light source in the photoreaction chamber, and the time of each group was recorded.

We took 2.5 mL of MMA monomer, added 21 mg TPO, initiated photopolymerization under 450 nm ultraviolet light for 15 min, then added 50 mg CsPbBr_3_ nanocrystal precipitate, stirred evenly, and added it into a specific mold to continue polymerization under an ultraviolet lamp. After polymerization for 30 min, solid CsPbBr_3_–PMMA luminescent resin was obtained, and strong green fluorescence of the resin was observed under ultraviolet light. CsPbCl_3_ luminescent resin and CsPbI_3_ luminescent resin were prepared by the same method.

### 2.3. Stability Test of CsPbX_3_@PMMA

By comparing the CsPbBr_3_@PMMA luminescent resin to the perovskite supernatant, the protective effect of PMMA on the perovskite was verified. The CsPbBr_3_–toluene solution and CsPbBr_3_@PMMA luminescent resin were added to appropriate amounts of water and ethanol, respectively, and their luminescence was observed.

The CsPbBr_3_@PMMA luminescent resin was heated. Then, the CsPbBr_3_@PMMA luminescent resin was placed on asbestos mesh, and the temperature was gradually increased by 30 °C. The image was acquired under the same parameters, and the same ultraviolet light was used to observe the luminous strength of the resin.

## 3. Results and Discussion

### 3.1. CsPbBr_3_@PMMA Structural Characterization

**Figure 1:**
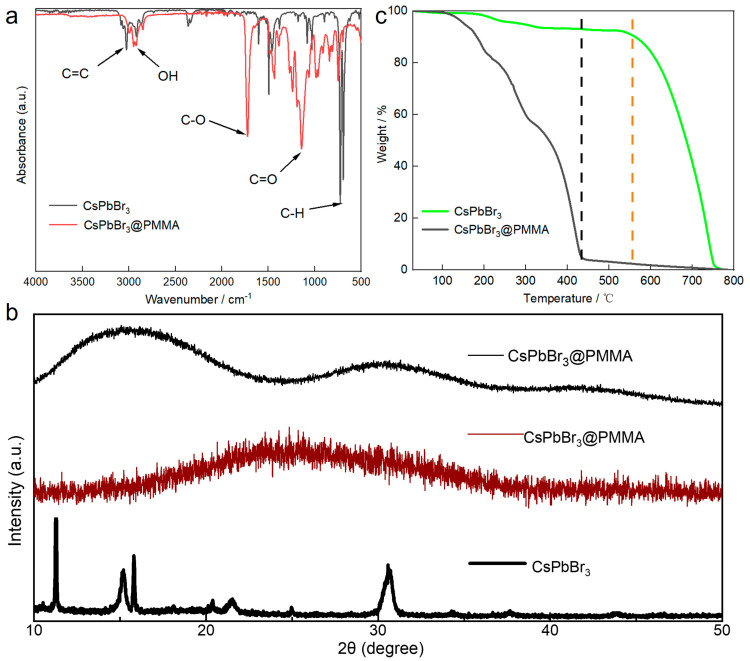
Structural characterization of CsPbBr_3_@PMMA: (**a**) Fourier transform infrared spectrum of perovskite in toluene solution and resin, (**b**) X−ray diffraction pattern of perovskite nanocrystals and perovskite resin (perovskite−PMMA bulk: black thin solid line (top), perovskite−PMMA powder: red thin solid line (middle), perovskite nanocrystal powder: black thick solid line (bottom)), (**c**) thermogravimetric analysis diagram of perovskite nanocrystals and perovskite resin.

The functional groups contained in the toluene solution and perovskite resin can be determined by Fourier transform infrared spectroscopy. Figure 1a shows that in the toluene solution, obvious carbon–hydrogen bond absorption peaks at 680 cm^−1^ on the benzene ring, carboxyl vibration absorption peaks at 1600 cm^−1^, hydroxyl absorption peaks at 3200 cm^−1^, and carbon–carbon double bond absorption peaks at 3350 cm^−1^ can be observed. It can be judged by the infrared spectrum that the perovskite nanocrystals contain toluene, oleyl amine, and oleic acid ligands. In the perovskite–PMMA resin, the ester group absorption peak at 1200 cm^−1^, the carboxyl group absorption peak at 1858 cm^−1^, and the hydroxyl group absorption peak at 3010 cm^−1^ can be observed. By Fourier infrared spectroscopy, it can be seen that in the PMMA resin, the perovskite resin also contains oleamine oleic acid ligands, and the PMMA resin will not destroy the chemical composition of the perovskite nanocrystals.

From the X-ray diffraction pattern of the perovskite (Figure 1b), it can be seen that the perovskite (CsPbBr_3_) nanocrystalline powder prepared by the high-temperature injection method is in a cubic crystal phase (black thick solid line). In the bulk resin of the perovskite (CsPbBr_3_)–PMMA, a relatively obvious carbon peak appeared at 15°, and the carbon peak appeared at 30° and 44°, indicating that the nanocrystalline perovskite also maintained a cubic phase (black fine solid line) in the PMMA resin. Therefore, PMMA will not destroy the spatial structure of the perovskite nanocrystals. After the perovskite–PMMA resin was ground into powder, due to the absorption of the resin voids and the destruction of the orthorhombic phase of the perovskite nanocrystals, no obvious cubic phase peak could be seen in the X-ray diffraction pattern. However, it could still be observed that the perovskite nanocrystals had a peak shape (red fine solid line), so it is inferred to be a cubic phase.

A thermogravimetric analysis test was performed on the centrifugally precipitated perovskite nanocrystal particles and the perovskite resin. The CsPbBr_3_@PMMA luminescent resin needs to be ground into a relatively fine powder. In nanocrystalline samples (Figure 1c), the temperature of metal salt volatilization is 556 °C (green solid line), while in resin, the temperature of metal salt volatilization is 442 °C (black solid line), and the test parameter is 20 °C/min. The reason for the above phenomenon is that the metal salts in the perovskite particles are aggregated, and the perovskite nanocrystals are not heated evenly, which leads to an increase in the time needed for metal melting and oxidation decomposition. The perovskite particles distributed in the resin have good dispersion, which leads to the rapid oxidation of metal salts and volatilization under high pressure air blowing, so the inflection point of the thermogravimetric analysis of the perovskite nanocrystalline luminescent resin occurs earlier than that of the pure perovskite nanocrystals.

### 3.2. CsPbBr_3_@PMMA Photophysical Properties

**Figure 2:**
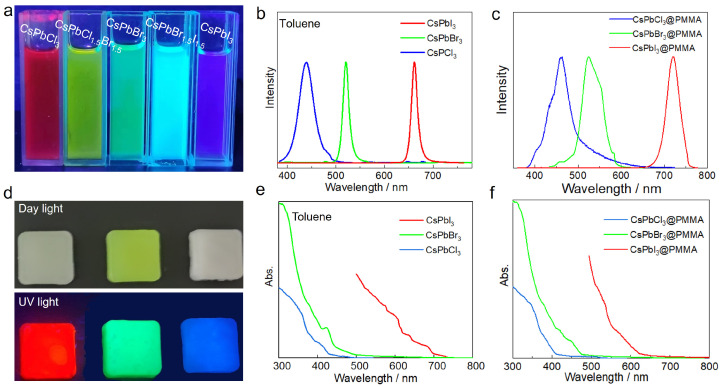
Photophysical properties of CsPbBr_3_@PMMA: (**a**) perovskite nanocrystals with different luminescence by changing halogen elements, (**b**) fluorescence spectra of CsPbX_3_–toluene solution, (**c**) fluorescence spectra of CsPbX_3_@PMMA resin, (**d**) perovskite luminescent resin under day light (upper) and ultraviolet light (lower), (**e**) ultraviolet absorption spectra of CsPbX_3_–toluene solution, (**f**) ultraviolet absorption spectra of CsPbX_3_@PMMA luminescent resin.

CsPbX_3_ perovskite nanocrystals with oleamine oleic acid as the ligand were synthesized by injection at high temperature. Different kinds of perovskite nanocrystalline toluene solutions were prepared by changing the proportion of halogen elements. Under ultraviolet light irradiation, the perovskite nanocrystals (from left to right, CsPbCl_3_, CsPbCl_1.5_Br_1.5_, CsPbBr_3_, CsPbBr_1.5_I_1.5_, and CsPbI_3_) can emit strong fluorescence of different colors (Figure 2a). Since pure chlorine was not prepared, the solution was mixed with trace (negligible) amounts of bromine, so the solution was red. By changing the halogen elements in the perovskite nanocrystals, the luminescence peak of perovskite nanocrystals can be adjusted to achieve perovskite nanocrystals with different luminescence colors. Then, 0.7% photoinitiator (TPO) was added to the PMMA solution. Under a daylight lamp, three kinds of resins were observed as opaque hard lumps (CsPbCl_3_, CsPbBr_3_, and CsPbI_3_, from left to right). The CsPbBr_3_@PMMA luminous resin was yellowish-green, and the rest were white. Upon irradiation with ultraviolet light at 365 nm, the three perovskites could be clearly observed to emit strong fluorescence (Figure 2d, top).

By observing the fluorescence luminescence spectra, CsPbCl_3_, CsPbBr_3_, and CsPbI_3_ had different luminescence peaks in the toluene solution, with the luminescence peaks located at 435 nm, 521 nm, and 685 nm, respectively (Figure 2b). In the PMMA resin, the luminescence peaks of the CsPbCl_3_ and CsPbBr_3_ perovskites were located at 430 nm and 515 nm, respectively. Compared with the perovskite nanocrystals stored in the toluene solution, there was a blueshift phenomenon, while the luminescence peak of CsPbI_3_ was located at 690 nm, showing a redshift phenomenon (Figure 2c). The reason for this phenomenon may be that the CsPbI_3_ perovskite nanocrystals have large particle sizes and their crystal phases are easily changed. The PMMA resin has a small porosity, and large perovskite nanocrystal particles are destroyed during the polymerization process, reducing the band gap, which provides a strong basis for the red shift of the nanocrystals [8].

According to the ultraviolet absorption spectrum (Figure 2e,f), the first exciton absorption peak of the perovskite nanocrystals in the toluene solution is approximately 510 nm, and different halogen perovskites have strong absorptions from 300 nm to 425 nm, so perovskites of three colors can be excited by using a light source of 365 nm. In PMMA, the first exciton absorption peak of the perovskites with different halogens is also located at approximately 510 nm, so the three perovskite resins can be effectively excited using a light source of 365 nm. The reasons for the difference in the absorption strength of the three kinds of perovskites are as follows: on the one hand, because the perovskite resin is thick and opaque, its reflected light and scattered light need to be measured in the ultraviolet absorption spectrum, and the surface smoothness of the resin also has a certain influence on the absorption strength; on the other hand, due to the different positions of the luminescent resin in the sample slot, the intensity of the luminescent resin in the detection of scattered light is different.

### 3.3. CsPbX_3_@PMMA Temperature Response and Stability Test

**Figure 3:**
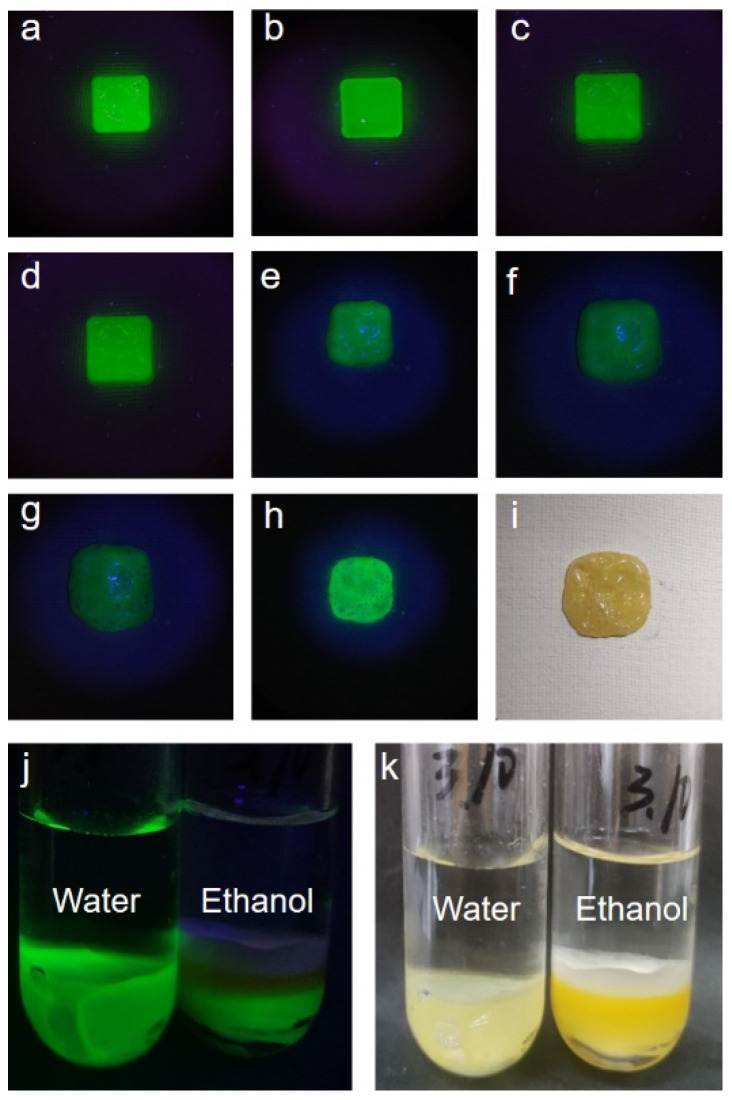
Temperature response and stability test of CsPbX_3_@PMMA luminescent resin: (**a**–**g**) luminescence change diagrams of the effect of temperature on perovskite, (**a**) 30 °C, (**b**) 60 °C, (**c**) 90 °C, (**d**) 120 °C, (**e**) 150 °C, (**f**) 180 °C, (**g**) 210 °C; (**h**) luminescence diagram of perovskite resin under UV lamp irradiation upon return to room temperature after heating; (**i**) perovskite resin under day light; (**j**) perovskite resin under ultraviolet light in water (left) and ethanol (right) solutions; (**k**) perovskite resin under day light in water (left) and ethanol (right) solutions.

For the temperature response test of perovskite, the conventional perovskite–toluene solution was completely extinguished when heated to 60 °C, and it no longer emitted light under ultraviolet irradiation after returning to room temperature. In the CsPbBr_3_@PMMA luminescent resin, when the perovskite resin was gradually heated to 150 °C, the luminescence of the perovskite resin began to weaken. After heating to 210 °C, the resin did not emit light (Figure 3a–g). The overall appearance was light green under ultraviolet light (Figure 3h) and yellow under a fluorescent lamp (Figure 3i). The resin began to melt at 120 °C, and the surface turned yellow at the same time. After returning to room temperature, the resin could emit strong fluorescence under irradiation with an ultraviolet lamp. The melting points of cesium bromide and lead bromide are 636 °C and 371 °C, respectively. Cesium lead bromide was heated at a high temperature for a long time, causing it to be in a semi-melted state. After returning to room temperature, the perovskite resin recrystallized. A cubic crystal phase was formed, and the photoluminescence properties were recovered. The same luminescent resin was heated to 210 °C three times to make it no longer luminescent, and the perovskite luminescence recovered after returning to room temperature, which indicates that the perovskite resin had a reversible temperature response. In addition, more bubbles were observed in the heated CsPbBr_3_@PMMA resin under fluorescent lamp irradiation, indicating that there were more voids in the PMMA resin. Under natural light, the perovskite resin turned yellow. It is speculated that there are two main reasons. On the one hand, a single PMMA resin has an obvious yellowing phenomenon after heating; on the other hand, the metal salt formed after perovskite agglomeration is also yellow in appearance.

To study the resistance of the perovskite–PMMA to polar solvents, ethanol and water were used as examples to observe their luminescence. After soaking the perovskite–PMMA in water and ethanol for 60 days, it still had a strong luminescence (Figure 3j). PMMA can be used as a protective material for the perovskites. We think that there are several main reasons: PMMA has good transparency, adhesion, ultraviolet resistance, convenient processing, and low hygroscopicity. In ethanol, due to the obvious depolymerization destruction effect of ethanol on PMMA, the depolymerization of PMMA cannot play a good role in protecting the perovskite, resulting in fluorescence quenching (Figure 3j, right). Compared with water (Figure 3k, left), ethanol has a stronger permeation effect on PMMA. Under natural light, it could be observed that ethanol agglomerated the upper layer of the perovskite nanocrystals, resulting in the resin turning yellow, and the yellow part did not emit light under ultraviolet light (Figure 3k, right).

### 3.4. CsPbX_3_@PMMA Application

**Figure 4:**
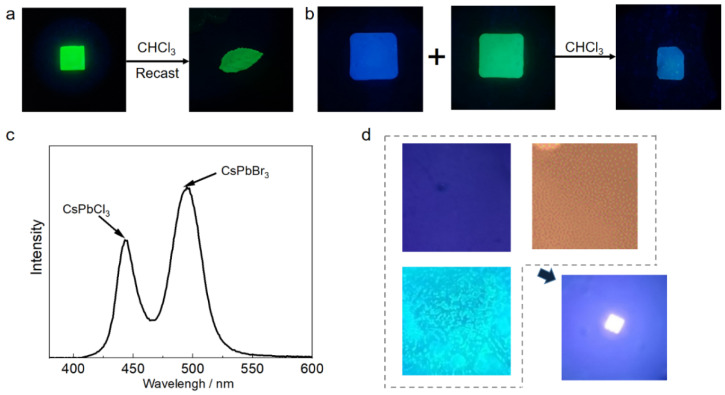
Application of CsPbX_3_@PMMA luminescent resin: (**a**) schematic diagram of dissolution and recasting of perovskite luminescent resin, (**b**) mixing diagram of two different color luminescent resins, (**c**) fluorescence spectrum diagram of the two resins mixed in (**b**), (**d**) after the three resins are overlapped, a white light source is prepared using ultraviolet light as the excitation light.

PMMA resin has high hardness, which is not conducive to the secondary processing of the CsPbX_3_@PMMA luminescent resin. In this paper, it was found that PMMA resin has good solubility in trichloromethane. The PMMA resin could be dissolved by using trichloromethane. When the trichloromethane was completely evaporated, the resin could be resolidified into a solid block. Here, taking the CsPbBr_3_@PMMA luminescent resin as an example, 2 g resin was dissolved in 1 mL trichloromethane, and it was completely dissolved by an ultrasonic machine for 30 min. After dissolution, it was added to a specific mold, and when the chloroform was completely volatilized, it could be reshaped into other shapes of perovskite resin (Figure 4a). For the dissolution and recasting of luminescent resin, resins of other colors can be prepared by mixing two different color resins. For example, blue CsPbCl_3_@PMMA and green CsPbBr_3_@PMMA luminescent resins were mixed to prepare a cyan luminescent resin (Figure 4b). According to the fluorescence spectrum (Figure 4c), the luminescence peaks of the resin were located at 425 nm and 490 nm, respectively, and the luminescence of the resin was the mixed light of two different colors rather than the luminescence after the exchange of two halogens, which indicates that PMMA had a good protective effect on the perovskite and can separate perovskites. Perovskites containing different halogens do not undergo ion exchange. On this basis, the perovskites with different colors were dissolved in chloroform and mixed with each other to prepare fluorescent coatings with different colors. The prepared coatings were evenly mixed, and they had the mixed light of different halogens instead of a single-color light after ion exchange.

Red, green, and blue are the three primary colors of light. Theoretically, white fluorescent resin can be prepared by mixing three different colors of perovskite resin in a certain proportion. We took three colors of luminescent resin, dissolved the three colors of resin in chloroform to obtain a viscous solution, evenly smeared the solution on three glass sheets, and observed the color under an ultraviolet lamp. Then, the three glass sheets were superimposed together in the order of blue, green, and red, and white luminescence was observed at the overlapping position of the three resins (Figure 4d).

## 4. Conclusions

In summary, CsPbX_3_@PMMA was prepared by using MMA as a monomer and TPO as a photoinitiator, which was initiated by ultraviolet light and encapsulated with the perovskite powder prepared by thermal implantation. PMMA has a good protective effect on the perovskite, and it still has strong luminescence after being stored in water for more than 60 days, which greatly improves the resistance of the perovskite to strong polar solvents. By changing the proportion of halogen elements in the perovskite resin, the color of the luminescent resin can be changed, and a variety of colors of CsPbX_3_@PMMA can be prepared. In this study, a series of polar solvent resistance and photophysical properties of CsPbX_3_@PMMA were tested. In addition, CsPbX_3_@PMMA was dissolved in chloroform to prepare an integrated molding resin. Finally, a white light source was prepared by superimposing the three primary colors of red, green, and blue CsPbX_3_@PMMA luminescent resin and using ultraviolet light as the excitation light. Theoretically, by only changing the proportion of luminescent resin, fluorescent coatings with luminescence in all visible light bands can be prepared, which has wide application prospects in the fields of architecture, industry, and transportation.

## Data Availability

The datasets supporting this article have been uploaded as part of the Supplementary Materials.

## Supplementary Materials

The following supporting information can be downloaded at: https://www.mdpi.com/article/10.3390/mi15091150/s1.
