# Supplementary material for: Preparation and Properties Study of CsPbX3@PMMA Luminescent Resin
Source: Micromachines (Basel). 2024 Sep 13;15(9):1150. doi: 10.3390/mi15091150 (PMC11434466; doi:10.3390/mi15091150)
Supplement: Supplementary file 1 [file micromachines-15-01150-s001.zip › micromachines-3113041-supplementary.pdf]

Supporting Information

Preparation and Properties Study of $CsPbX_3$@PMMA Luminescent Resin

1 Experimental reagents

Reagent	Purity	Supplier
$PbBr_2$	AR	Meryer (Shanghai) Biochemical Technology Co., Ltd.
CsBr	AR	Meryer (Shanghai) Biochemical Technology Co., Ltd.
N,N-Dimethylformamide	AR	Sinopharm Chemical Reagent limited corporation
Oleylamine	AR	Energy Chemical Technologies Co., Ltd.
Oleic acid	AR	Energy Chemical Technologies Co., Ltd.
Methyl methacrylate	AR	Energy Chemical Technologies Co., Ltd.
2,4,6-trimethylbenzoyl phosphine oxide	AR	Energy Chemical Technologies Co., Ltd.
Trichloromethane	AR	Sinopharm Chemical Reagent limited corporation
Toluene	AR	Sinopharm Chemical Reagent limited corporation
PbI_2	AR	Meryer (Shanghai) Biochemical Technology Co., Ltd.
$PbCl_2$	AR	Meryer (Shanghai) Biochemical Technology Co., Ltd.
Caesium carbonate	AR	Meryer (Shanghai) Biochemical Technology Co., Ltd.
Octadecene	AR	Sinopharm Chemical Reagent limited corporation

2. Experimental instruments

Instrument	Type	Supplier
Electronic balance	FA 2004	Shanghai Sunny Hengping Scientific Instrument Co., Ltd.
Electronic analytical balance	Ex 125DZH	OHAUS Instruments (Changzhou) Co.,Ltd.
Electric blast drying oven	GZX-9140MBE	Shanghai Boxun Medical Biological Instrument Co.,Ltd.
Magnetic stirrer	C-MAG HS7	IKA-Werke GmbH & CO. KG Janke & Kunkel-Str (Guangzhou)
Ultrasonic cleaner	KQ-250DE	Ultrasonic Instrument (Kunshan) Co., Ltd.
High speed centrifuge	Cence H1850	Hunan Xiangyi Laboratory Instrument Development Co., Ltd.
Pipette	Eppendorf	Eppendorf China Co., Ltd.
Fluorescence photometer	RF-6000	Shimadzu (China) Co., Ltd.
Luminescence spectrometer	BR-527	German Huibolong Precision Instruments Co., Ltd.
UV-visible spectrophotometer	UV-2600	Shimadzu (China) Co., Ltd.
X-ray diffractometer	Empyrean	Netherlands Malvern Panalytical
Fourier transform infrared spectrometer	INVENIO-R	Bruker Physik-AG Co., Ltd.
Thermogravimetric analyzer	TGA-55	TA Instruments
Steady-state and transient modular luminescence spectrometer	QuantaMaster 8000	HORIBA, Ltd.

3. Experimental method

3.1 Fluorescence photometer and luminescence spectrometer test

Dissolve the sample to be tested with the corresponding solvent, dilute it to an appropriate concentration, and then put the solution into a quartz cuvette. Set the parameters of the luminescence spectrometer so that the excitation wavelength is 365 nm and the test wavelength range is 380-650 nm. For solid samples, use a luminescence spectrometer to perform luminescence spectrum testing. Prepare the sample to be tested to a suitable size and place it on the sample holder. Set the excitation wavelength to 365 nm and the test wavelength to 380-650 nm.

3.2 UV-visible absorption spectroscopy test

Dissolve the sample to be tested with the corresponding solvent, dilute it to a suitable concentration, then put the solution into a quartz cuvette, set the parameters of the UV-visible absorption spectrometer, and the test wavelength is 300-700 nm. For solid samples, use a UV absorption

spectrophotometer for testing. Place the solid sample on the sample holder and test the same parameters as the solution sample.

3.3 Luminous life test

The QuantaMaster8000 steady-state transient modular luminescence spectrometer was used to test the luminescence emission lifetime of the sample. For solution samples, the sample to be tested needs to be diluted to an appropriate concentration, put into a cuvette, and the luminescence spectrum is measured to determine the position of the luminescence peak. Then select a laser with an appropriate wavelength, and test the luminescence emission lifetime at the luminescence peak. For solid samples, they can be placed directly on the sample holder. The solid sample is tested for scattered light and refracted light. The surface must be as flat as possible, and the test conditions are the same as the solution sample.

3.4 Luminescence quantum yield test

Luminescence quantum yield is defined as the ratio of the number of photons emitted by a luminescent substance after absorbing light to the number of photons of excitation light absorbed. Normally, the value of luminescence quantum yield is less than 1. The larger the value of the luminescence quantum yield, the stronger the fluorescence of the compound. In this article, the test data are all tested using a steady-state transient modular luminescence spectrometer, using a 365 nm light source for excitation, and an R928 single-photon detector to collect data. Test the luminescence quantum yield of blank samples, solution samples and solid samples.

3.5 Fourier transform infrared spectroscopy test

The testing methods of Fourier transform infrared spectroscopy vary depending on the state of the sample. For liquid samples, the sample needs to be spread onto a quartz plate for testing. For solid powder samples, the sample needs to be added to the fully ground potassium bromide powder and ground evenly. After drying under an infrared lamp, tablets were pressed and tested. For block samples, the block needs to be placed above the test hole and compressed using a tablet pressing device. The test range is 400~4000 cm^{-1}.

3.6 Thermogravimetric analysis

Thermogravimetric analysis is an analytical technique that measures the total mass change of the sample to be tested by continuously increasing the temperature according to the different decomposition temperatures of substances. It is often used to study the thermal stability and composition of materials. In this article, the solid sample is fully dried, placed in a previously peeled crucible, and oxygen is introduced to cause oxidation and decomposition for testing. For bulk samples, they first need to be ground into fine powder.

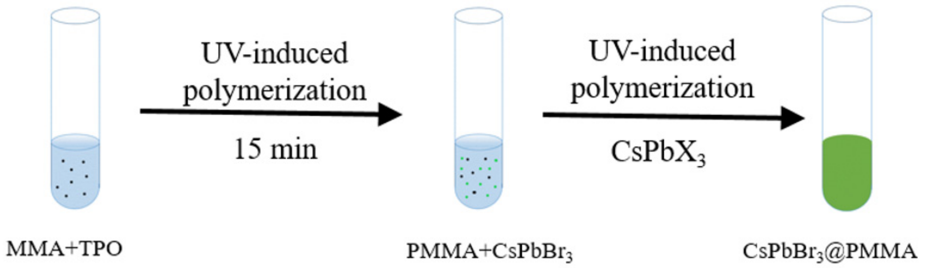

Figure S1. Schematic diagram of preparation of $CsPbBr_3$@PMMA luminescent resin.
